# Associations between objective hearing function and subjective views of aging

**DOI:** 10.1007/s10433-025-00868-8

**Published:** 2025-07-16

**Authors:** Jana Koch, Brooke Brady, Lidan Zheng, Kaarin J. Anstey

**Affiliations:** 1 School of Psychology, University of New South Wales Sydney, High Street, Kensington, NSW 2052 Australia; 2 Neuroscience Research Australia, Randwick, Australia; 3 UNSW Aging Futures Institute, UNSW Sydney, Kensington, NSW Australia

**Keywords:** Hearing loss, Expectations regarding aging, Self-perceptions of aging, Views on aging

## Abstract

Hearing loss, a common age-related health condition, has been linked to adverse health outcomes, including changes in social participation and cognitive function. As subjective views of aging are influenced by changes in health and functional abilities, we hypothesized that people with poor hearing would exhibit less favorable generalized and personal Views of Aging. Additionally, we explored whether these associations varied by age. Data were analyzed from 148 participants (aged 40–84) who completed an app-based research study: Labs without Walls. Participants completed a validated, app-based hearing task, and a pure-tone average was calculated in the better-hearing ear. Generalized Views of Aging were measured using the Expectations Regarding Aging Scale (with subscales on physical health, mental health, and cognitive function). Personal Views of Aging were measured using the Self-perceptions of Aging Scale. Structural equation modeling was conducted to explore the relative contributions of hearing function to Views of Aging constructs while controlling for chronological age, sex-at-birth, sociodemographic status, loneliness, and cognition. Cross sectionally, poorer hearing was associated with negative age expectations regarding maintaining physical health and with negative self-perceptions of aging. Contrary to our hypothesis, hearing function did not predict age expectations about mental health or cognitive function. No significant age moderation effects were observed. Overall, these findings offer preliminary evidence for distinct associations between hearing and individual Views of Aging constructs and domains. The novel insight into the association between objectively measured hearing and Views of Aging highlights the importance of addressing hearing health early in the aging process to prevent negative outcomes linked to Views of Aging.

**Supplementary Information:**

The online version contains supplementary material available at 10.1007/s10433-025-00868-8.

## Introduction

Age-related hearing loss not only symbolizes a decline in health but also serves as a constant reminder of the inevitability of aging (Wallhagen 2009). Additionally, the social stigma surrounding hearing loss often leads to challenges with self-image, as people with hearing loss fear being perceived as old, fragile, or less capable (Wallhagen 2009). This stigma might influence how individuals view their own aging, while reinforcing negative attitudes and affecting their subjective experience of growing older (Diehl et al. 2014). Views of Aging is an umbrella term used to describe how individuals interpret, understand, and refer to the aging experience (Shrira et al. 2022). Research interest in Views of Aging has grown exponentially in the past few decades (Diehl et al. 2014; Shrira et al. 2022), driven at least in part by the global rise in population aging (World Health Organization 2020). With this demographic shift, however, there has also been an increase in age-related health conditions. Hearing loss (HL) is common, with around 65% of those over the age of 60 experiencing it (World Health Organization 2021). Difficulties can range from mild (i.e., trouble following conversation in loud surrounding), moderate (i.e., trouble following conversation in quiet surrounding), severe (i.e., need loud speech to hear in quiet surrounding and has great difficulty with noise), to profound (i.e., unable to hear even loud or shouted speech in quiet surrounding) (Humes 2019). Hearing loss can have significant physical, cognitive, mental, and social consequences and has been linked to poorer quality of life and well-being (Tsimpida et al. 2022). For instance, hearing impairment is related to an increased risk of depression (Lawrence et al. 2020) and loneliness (Huang et al. 2021). Furthermore, the 2024 Lancet Commission report on dementia prevention lists hearing loss as a major modifiable risk factor for dementia, which, if treated, may delay the onset of cognitive decline and dementia (Livingston et al. 2024). While it is well-documented that hearing loss can have adverse effects on health (Henderson et al. 2024), psychosocial (Lawrence et al. 2020), and cognitive (Livingston et al. 2024) well-being, this study explores the associations between hearing function and subjective views of aging (VoA).

### Generalized and personal views of aging

There are several widely used measures of VoA in the literature, each measuring a slightly different aspect of subjective VoA. In a recent effort to categorize these, Shrira et al. (2022) proposed a conceptual hierarchy, dividing each construct into (1) generalized VoA, or (2) personal VoA (Shrira et al. 2022). Generalized VoA reflect societal beliefs of aging, or age-related stereotypes, which are shaped by underlying cultural and family beliefs, health, as well as subtle but consistent age-related stereotypes present in everyday life (Shrira et al. 2022). These factors often influence thoughts and behaviors on an unconscious level (Diehl et al. 2014; Kornadt et al. 2016) and, according to the stereotype embodiment theory, contribute over time to self-perceptions of aging, or personal VoA (Levy 2009). Personal VoA reflect an individual’s interpretation and experience of their own aging process (Diehl & Wahl 2024; Kornadt et al. 2020; Shrira et al. 2022).

### Hearing function as antecedent for views of aging: current evidence

It is anticipated, from a theoretical perspective, that declines in physical health or the presence of illness (i.e., hearing loss) are likely to contribute to more negative VoA (Kornadt et al. 2020). As well, theories of aging and human development emphasize that age-related changes are fundamental to VoA (Diehl et al. 2014). Here, we briefly outline common measures of generalized and personal VoA and summarize available evidence for hearing as an antecedent to each construct.

Most empirical attention has been given to the relationship between hearing function and constructs reflecting personal VoA, showing associations with an older subjective age (Schroyen et al. 2020; for similar results, see: Langballe et al. 2023) and greater awareness of age-related losses in cognitive function and physical health domains (Wettstein et al. 2023b). To date, the most studied personal VoA measure in relation to hearing function is *Self-perceptions of aging (SPA)* (operationalized here as the Attitudes Towards Own Aging Scale, Lawton 1975), which refers to beliefs an individual holds about their own aging (Kornadt et al. 2020). SPA is a unidirectional construct as it categorizes age perceptions on a spectrum from positive to negative, without considering different life and behavioral domains (Shrira et al. 2022).

Four studies have investigated the relationship between hearing function and SPA, yielding mixed results (Jang et al. 2004; Kim et al. 2012; Nakagawa et al. 2018; Wettstein et al. 2023a). Two cross-sectional studies found no significant association (Jang et al. 2004; Kim et al. 2012), while another suggest culture-specific associations, with an association observed in a Japanese subsample, but not an American one (Nakagawa et al. 2018). Additionally, no longitudinal relationship was found by Wettstein and colleagues, the only study to use an objective measure of hearing (Wettstein et al. 2023a). These inconsistencies could be due to methodological differences. For example, while most studies used a self-rated measure of hearing (Jang et al. 2004; Kim et al. 2012; Nakagawa et al. 2018), only one study has included an objective measure of hearing function (Wettstein et al. 2023a). More research is needed that utilizes an objective measure of hearing to clarify how objective changes in sensory function relate to VoA. Additionally, a decline in health, such as the onset of hearing loss, in midlife (i.e., 40 +) is often experienced as an ‘off-time’ experience and thus might have a differentiated influence on VoA compared to older adults (i.e., 70 +) (Kornadt et al. 2020; consistent with: Wettstein et al. 2023b). However, all existing studies on SPA and hearing have focused exclusively on older adults 60 + (Jang et al. 2004; Kim et al. 2012; Nakagawa et al. 2018; Wettstein et al. 2023a), limiting insight into how these associations might vary across different life stages (i.e., middle-age and old age).

*Expectations regarding aging (ERA)*, a commonly measured construct of generalized VoA, captures expectations of ‘healthy aging for both self and others’ (Sarkisian et al. 2005). Unlike other generalized VoA constructs such as attitudes toward aging or essentialist beliefs, ERA specifically focuses on future expectations about aging across multiple domains (i.e., mental health, cognitive function, and physical health). ERA has been linked to health behaviors, such as help-seeking (Sarkisian et al. 2002), which is particularly relevant in the context of hearing loss. Given that individuals with hearing loss already under-utilize healthcare services (Knoetze et al. 2023), those with more negative aging expectations may be even less likely to seek support. Furthermore, examining both personal (SPA) and generalized VoA (ERA) within the same theoretical and statistical model provides an opportunity to extend the current literature. According to theories of aging and human development (Diehl et al. 2014; Levy 2009), generalized VoA shape personal VoA. In this context, negative age expectations could, over time, develop into more negative self-perceptions of aging. Thus, considering ERA may help account for the inconsistencies in the relationship between hearing loss and SPA found in previous studies. To our knowledge, however, no study has yet examined the association between hearing function and ERA.

### Health status and views of aging: a reciprocal relationship

It is important to acknowledge that VoA has been identified as both an outcome *and* predictor of health status (Kornadt et al. 2020). In other words, while a decline in physical health or the presence of illness is likely to contribute to more negative VoA (Schönstein et al. 2021; Stephan et al. 2018), negative VoA, in turn, also contribute to adverse health outcomes over time, including longevity (i.e., self-perceptions of aging, Levy et al., 2002), physical health (i.e., self-perceptions of aging, Sargent-Cox et al., 2012), and well-being (i.e., awareness of age-related change, Sabatini et al. 2020). These pathways are largely explained by the stereotype embodiment theory (Levy 2009), which posits that internalized negative age stereotypes can influence behavioral, psychological and physical outcomes over time (Levy 2009). In the context of hearing loss, empirical evidence supports a reciprocal relationship between hearing and VoA, showing that negative VoA can also lead to poorer hearing function over time (Levy et al. 2006; Stephan et al. 2022). Although the cross-sectional nature of the current study prevents us from directly testing the direction of relationships between hearing function and VoA, it is important to explore how hearing function influences VoA, as negative VoA, in turn, could reduce engagement in health behaviors (i.e., hearing aid use), with potential long-term impacts on well-being and cognitive outcomes.

### The present study

Therefore, the aim of this study is to provide preliminary evidence for the role of objectively measured hearing function as a predictor for generalized VoA (Expectations Regarding Aging) and personal VoA (Self-perceptions of Aging). We hypothesize that poorer hearing will be associated with (a) more negative expectations regarding aging across all three domains (mental health, cognitive function, physical health) and more (b) negative self-perceptions of aging. As described above, these constructs are distinct from one another as they are used to evaluate different parts of the aging experience (Kornadt et al. 2020), thus they might differ regarding the strength of their associations with hearing function. Given the inclusion of adults aged 40 and above, we will also explore whether age moderates the relationship between hearing function and VoA.

## Methods

### Study design

This study used data from Labs without Walls (LwW), an app-based study of life course aging, aiming to understand how self-perceptions of aging and gender can be impacted by age and other factors (Brady et al. 2023). LwW adapted a multi-timescale measurement burst design, where participants first completed a baseline questionnaire via the LwW research app. Over the following eight weeks, they completed repeated short questionnaires (e.g., a 7-day ‘Mood Survey’, assessing daily affect) and game-like active tasks (e.g., app-based hearing task and cognitive tests) via the same research app on their iPhones. The study was conducted from February 2022 to January 2023 and included a total of 225 individuals living in Australia, aged 18–85, who owned an iPhone, had access to the internet and were willing to download the LwW research app and wear an Apple Watch for the duration of the study. Participants were recruited via social media platforms such as Facebook, professional media call outs, and the Neuroscience Research Australia Healthy Research Volunteer Registry.

For this current study, we used a cross-sectional dataset from the LwW baseline questionnaire (week 1, including sociodemographic and VoA measures), and results from the app-based hearing task collected in weeks three and seven. As age-related hearing loss typically becomes more prevalent and relevant in middle and older adulthood (World Health Organization 2021), we excluded participants under the age of 40 from this current analysis (for a similar approach, see: Wettstein et al. 2023b). Additional eligibility criteria for the present study were that participants had completed the hearing task on at least one of the two time points (week 3 or week 7) and have valid data for the frequencies used to calculate the pure-tone average (PTA). Ethics approval was obtained from the University of New South Wales Human Research Ethics Committee (HC200792), and all participants provided informed e-consent within the research app (Brady et al. 2023).

### Measures

#### Sociodemographic variables

Sociodemographic variables included chronological age in years, and sex assigned at birth (male, female, another term). No participants in this sample reported a sex-at-birth other than male or female. Socioeconomic status consisted of a composite score of total years of education (in years) and household income per week (0 =  < $300; 1 = $300–575; 2 = $575–1075; 3 = $1075–1700; 4 = $1700–2400; 5 =  > $2400). Both variables were treated in their raw form, with the total years of education and income category scores summed to create a composite score. Higher composite scores indicate greater socioeconomic advantage. Loneliness was measured on the 3-item UCLA loneliness scale (Hughes et al., 2004). Participants were asked to respond on a 3-point Likert scale. A total loneliness score was calculated by summing scores for each item. Scores range from 3 to 9, with higher scores indicating increased loneliness. Cronbach’s alpha for this scale in the present sample was 0.87. Employment status (employed, unemployed, and retired), relationship status (married, in relationship, single, widowed, and divorced), and ethnicity (White, Northeast Asian, South and Central Asian, West Asian, Hispanic/Latin American, Jewish, and Arab) were included as additional variables to describe the sample.

#### Hearing function

Pure-tone audiometry using an audiometer has been considered the gold standard and the most common method for measuring HL (Humes 2019). However, more cost and time-effective alternatives are available, including self-rated hearing questionnaires (Kim et al. 2012) and free-to-use mobile phone applications that assess hearing function (Almufarrij et al. 2023). App-based hearing tests are particularly promising, as they can enable the large-scale collection of data regarding objective hearing function in longitudinal, population-based studies that were previously hindered by the logistics of in-person hearing tests or limitations of self-rated hearing questionnaires (Fischer & Kleen 2021).

This study assessed hearing function using the dBHL Tone Audiometry task from the Apple ResearchKit Framework (http://researchkit.org/) administered on the LwW app. All participants received Apple wired EarPods (model number MMTN2FE/A) for consistent hearing task delivery. Regardless of their usual hearing aid use, all participants were prompted to use the EarPods, therefore, the hearing function assessed in this study captures uncorrected hearing. The LwW app directed participants to maximize their volume level to 100% for optimal data collection conditions and enhance the hearing data quality. Additionally, the task captured environmental noise before the hearing task, and participants only began once they were in a sufficiently quiet location.

During the hearing task, participants were presented with a series of tones and tapped a button on the iPhone screen to indicate when they heard each tone. The hearing task utilized the Hughson–Westlake method to record the lowest decibel hearing level (dBHL), requiring the participants to correctly detect the tone played at each frequency 50% of the time. A four-frequency PTA of the better-hearing ear was calculated with frequencies 0.5, 1, 2, and 4 kHz. The degree of HL was classified using the World Health Organization grades (Humes 2019) (see Supplementary Table S1). The first valid PTA from either week 3 or 7 will be used for analysis. When participants did not respond to any of the tones at a certain frequency, the data were marked as 100,000 and treated as missing. The app-based hearing task in LwW has been validated against face-to-face lab-based pure-tone audiometry using a SHOEBOX tablet-based audiometer among a subsample of LwW participants (Zhou et al. 2025). The agreement between face-to-face audiometry and the app-based hearing task across frequencies ranged from moderate to excellent, with intra-class correlations between 0.56 and 0.96 (*p* < 0.001) (Zhou et al. 2025).

#### Generalized views of aging

Participants’ generalized VoA were measured by the 12-item Expectations Regarding Aging Scale (ERA) (Sarkisian et al. 2005). The ERA scale has three subscales: expectations regarding physical health (i.e., ‘The human body is like a car: it gets worn out’), mental health (i.e., ‘It’s normal to be depressed when you are old’), and cognitive function (i.e., ‘Forgetfulness is a natural occurrence just from growing old’). Participants respond to each item using a 4-point Likert-type scale (1 = Definitely true, 2 = Somewhat true, 3 = Somewhat false, 4 = Definitely false). Subscale scores were calculated and rescaled following author instructions (Sarkisian et al. 2005). Cronbach’s alpha for the ERA physical health, mental health, and cognitive function subscales in the present sample were 0.79, 0.71, and 0.84, respectively.

#### Personal views of aging

Personal VoA were measured using the 5-item Attitudes toward own Aging subscale of the Philadelphia Geriatric Morale Scale, a common measure of self-perceptions of aging (SPA) (Lawton 1975). The scale measures a personal evaluation of one’s own aging process with items such as ‘Things keep getting worse as I get older’ or ‘I have as much energy as I did last year’. Participants indicate to what extent they agree with each item using a 4-point Likert scale (1 = Strongly disagree, 2 = Disagree, 3 = Agree, 4 = Strongly agree). After reverse coding two items so that all items are measuring negative perceptions, a total score was calculated. Higher scores indicate more negative self-perceptions of aging. In this sample, Cronbach’s alpha of this scale was 0.74.

#### Cognitive function

Cognitive function was assessed using an app-based version of the Trail Making Test B from the Apple ResearchKit Framework (http://researchkit.org/) administered on the LwW app in week 1. This test is intended to measure cognitive flexibility, alternating attention, visual search and processing speed. Participants are instructed to alternate between tapping circles labeled with numbers and those labeled with letters (i.e., 1, A, 2, B, 3, C, etc.). The test consists of a total of 13 circles that participants needed to connect (7 numbers, 6 letters). The LwW app recorded the total number of errors and completion time. The app-based Trail Making Test B used in LwW has been validated against a face-to-face lab-based alternative (i.e., pen-and-paper Trail Making Test) in a subsample of LwW participants (Zhou et al. 2025). The correlations between the pen-and-paper and the app-based Trail Making Test B were strongly positively correlated (r = 0.78, *p* < 0.001) (Zhou et al. 2025). For this analysis, we used the total time (in seconds) that participants took to complete the task as a measure of cognitive function, as this is the traditional scoring method for Trail Making B, capturing errors through increased completion time (Reitan & Wolfson 1985).

### Data analysis

Descriptive statistics were used to provide an overview of the key sample characteristics, while chi-square and t-tests compared demographics between those with and without hearing loss. Bivariate correlation coefficients were calculated to examine the associations between variables of interest. To evaluate the contribution of hearing function on VoA constructs, structural equation modeling (SEM) was conducted. The benefit of using SEM compared with regression analyses is the use of latent variables and its ability to account for measurement errors associated with observed variables. By integrating the reliability statistic, such as Cronbach’s alpha of the validated ERA and SPA scales, SEM provides more accurate estimates of the theoretical pathways of interest (Bollen 1989). Fitting a SEM to our data also allows us to examine all pathways of interest within one model, rather than conducting multiple separate regression analyses, hence, reducing the risk of Type II errors. The proposed model included one exogenous variable (PTA) and four endogenous variables (ERA physical health, ERA cognitive function, ERA mental health, SPA) (Fig. 2). Rather than having one latent variable for expectations regarding aging, we included each of the subscales as separate latent variables to get a better understanding of the unique contributions of hearing function on these subscales. To account for factors that might affect both hearing function and VoA, chronological age, sex, socioeconomic status, loneliness, and cognitive function were considered as potential covariates. To reduce the number of variables in the final SEM model, we inspected bivariate correlation coefficients and only included sociodemographic control variables if they were significantly correlated with either PTA or the VoA measures (i.e., chronological age, sex, and loneliness). Chronological age and sex were entered as observed variables, whereas we created a latent variable for loneliness, measured by the three items of the UCLA scale (see Supplementary Figure S1). Finally, we included an interaction term (PTA X chronological age) to explore any age effects within our results.

Model fit was deemed adequate with chi-square goodness of fit statistic above p > 0.05 ( ﻿χ^2^; Schumacker & Lomax 2010), root mean square error of approximation value below 0.05 (LO90 = 0.00, PCLOSE > 0.05) (RMSEA; Browne & Cudeck 1992), standardized mean square residual below 0.06 (SRMR; Hu & Bentler, 1999), the Tucker–Lewis Index above 0.95 (Tucker & Lewis 1973), and comparative fit index above 0.95 (CFI; Bentler 1990). The model was evaluated based on the suggested modification indices for optimization and best model fit. We included covariances between factors and/or error terms only if they made theoretical sense and statistically improved the overall model.

To account for missing data, Expectation Maximization (EM) estimation was used for cases where less than 20% of the variables were missing (Enders 2001). Specifically, five cases had missing data on the Trail Making Task (3.4% of the sample), and one case had missing data on the UCLA scale (0.7% of the sample), both of which were imputed using EM. A p value of < 0.05 was used to determine statistical significance. All statistical analyses were conducted using IBM SPSS Statistics v28, Jamovi v2.4, and AMOS version 28. This analysis was pre-registered on Open Science Framework and its hypotheses, data analysis plan, measures collected, and sample size rationale are publicly available (10.17605/OSF.IO/YCS38).

## Results

### Participant characteristics

Of the 225 LwW participants, 167 were aged 40 and over. Of those, 19 participants were excluded as they had no or incomplete hearing task data (i.e., > 20% missing). The final sample consisted of 148 participants aged between 40 and 84 (M age = 62.11; SD = 11.5), of which the majority was female (60.8%) and White (88.5%). Supplementary Figure S3 illustrates the participant inclusion/exclusion process. Based on the validated, app-based hearing task, 52 participants (35.1%) had some level of hearing loss (20.9% mild hearing loss, 12.2% moderate hearing loss, 2% severe hearing loss), with PTA ranging from 0 to 65 (higher scores indicate worse hearing function). The demographic characteristics and group comparisons are presented in Table 1.

**Table 1:** Demographic characteristics of the sample

Demographics	Total sample (*N* = 148)	With hearing loss (*n* = 52)	Without hearing loss (*n* = 96)	Test statistics, *p* value
Age, mean (SD)	62.1 (11.5)	68.3 (9.4)	58.8 (11.2)	t(146) = −5.2, *p* <.001
Age groups, n (%)				χ^2^(1) = 15.3, *p* <.001
40–64	86 (58.1)	19 (22.1)	67 (77.9)	
65 +	62 (41.9)	33 (53.2)	29 (46.8)	
Sex, %				χ^2^(1) = 2.7, *p* =.103
Female	90 (60.8)	27 (51.9)	63 (65.6)	
Male	58 (39.2)	25 (48.1)	33 (34.4)	
Years of education, years (SD)	18.9 (4.3)	19.4 (4.7)	18.7 (4.1)	t(92.7) = -.10, *p* =.321
Ethnicity, n				χ^2^(6) = 7.4, *p* =.282
White	131	51	80	
Northeast Asian	3	–	3	
South and Central Asian	9	1	8	
Arab/West Asian	1	–	1	
Hispanic/Latin American	2	–	2	
Jewish	1	–	1	
Arab/West Asian–White	1	–	1	
Employment status, %				χ^2^(2) = 9.3, *p* =.010
Employed	86 (58.1)	22 (42.3)	64 (66.7)	
Unemployed	4 (2.7)	1 (1.9)	3 (3.1)	
Retired	58 (39.2)	29 (55.8)	29 (30.2)	
Household income (per week), n (%)				χ^2^(6) = 7.1, *p* =.315
< $300	2 (1.4)	1 (1.9)	1 (1)	
$300–575	11 (7.4)	6 (11.5)	5 (5.2)	
$575–1075	28 (18.9)	10 (19.2)	18 (18.8)	
$1075–1700	29 (19.6)	14 (26.9)	15 (15.6)	
$1700–2400	21 (14.2)	6 (11.5)	15 (15.6)	
> $2400	49 (33.1)	12 (23.1)	37 (38.5)	
Don’t know				
Relationship status, n (%)				χ^2^(4) = 5.6, *p* =.229
Married	91 (61.5)	31 (59.6)	60 (62.5)	
In relationship	15 (10.1)	6 (11.5)	9 (9.4)	
Single	22 (14.9)	5 (9.6)	17 (17.7)	
Widowed	9 (6.1)	6 (11.5)	2 (3.1)	
Divorced	11 (7.4)	4 (7.7)	7 (7.3)	
Hearing characteristics, n (%)				
No hearing loss	96 (64.9)	0	96 (100)	
Hearing loss	52 (35.1)	52 (100)	0	
Mild hearing loss	31 (20.9)	31 (59.6)	–	
Moderate hearing loss	18 (12.2)	18 (34.6)	–	
Severe hearing loss	3 (2)	3 (5.8)	–	
Pure-Tone Average, dBHL (SD)	19.8 (12.7)	33.9 (10)	12.2 (5.3)	t(146) = −17.3, *p* <.001

dBHL = decibel hearing level; No hearing loss = 0–20 dBHL, Mild hearing loss = 21–34 dBHL, Moderate hearing loss = 35–49 dBHL, Severe hearing loss =  > 50 dBHL; SD = Standard deviation. χ^2^ = Pearson Chi-Square test; *t* = Independent sample *t* test

### Bivariate analysis

Variable means, standard deviations, and bivariate correlation coefficients are reported in Table 2. We found that hearing function was negatively correlated with expectations regarding aging on the physical health subscale, and the cognitive function subscale. However, correlations were not significant between hearing function and expectations regarding mental health or self-perceptions of aging.

**Table 2:** Means, standard deviations, and correlations of study variables of interest

		*M*	*SD*	1	2	3	4	5	6	7	8	9	10
*Independent variable*													
1	Hearing function	19.8	12.7										
*Dependent variables*													
2	ERA, cognitive function	41.5	21.8	−.23^**^									
3	ERA, physical health	42.8	22.2	−.27^**^	.44^***^								
4	ERA, mental health	67.8	20.5	−.08	.49^***^	.53^***^							
5	SPA	9.3	2.5	−.01	.29^***^	.50^***^	.44^***^						
*Control variables*													
6	Chronological age	62.11	11.5	.52^**^	−.24^**^	−.09	.003	.01					
7	Sex	–	–	−.18^*^	.22^**^	.20*	.28^***^	.07	−.17^*^				
8	Socioeconomic status	22.3	4.2	.01	.13	.02	−.02	−.01	−.15	.03			
9	Loneliness	1.4	1.7	−.05	−.13	−.18^*^	−.37^***^	−.49^***^	−.15	.06	.05		
10	Trail Making B	22.5	19.8	.07	−.14	.01	−.06	.08	.24^**^	−.22^*^	−.17^*^	−.09	

^*^p < 0.05

^**^p < 0.01

^***^p < 0.001. ERA = expectations regarding aging. SPA = self-perceptions of aging. Sex (0, male; 1, female)

### Structural equation model: development of the final model

**Fig. 1:**
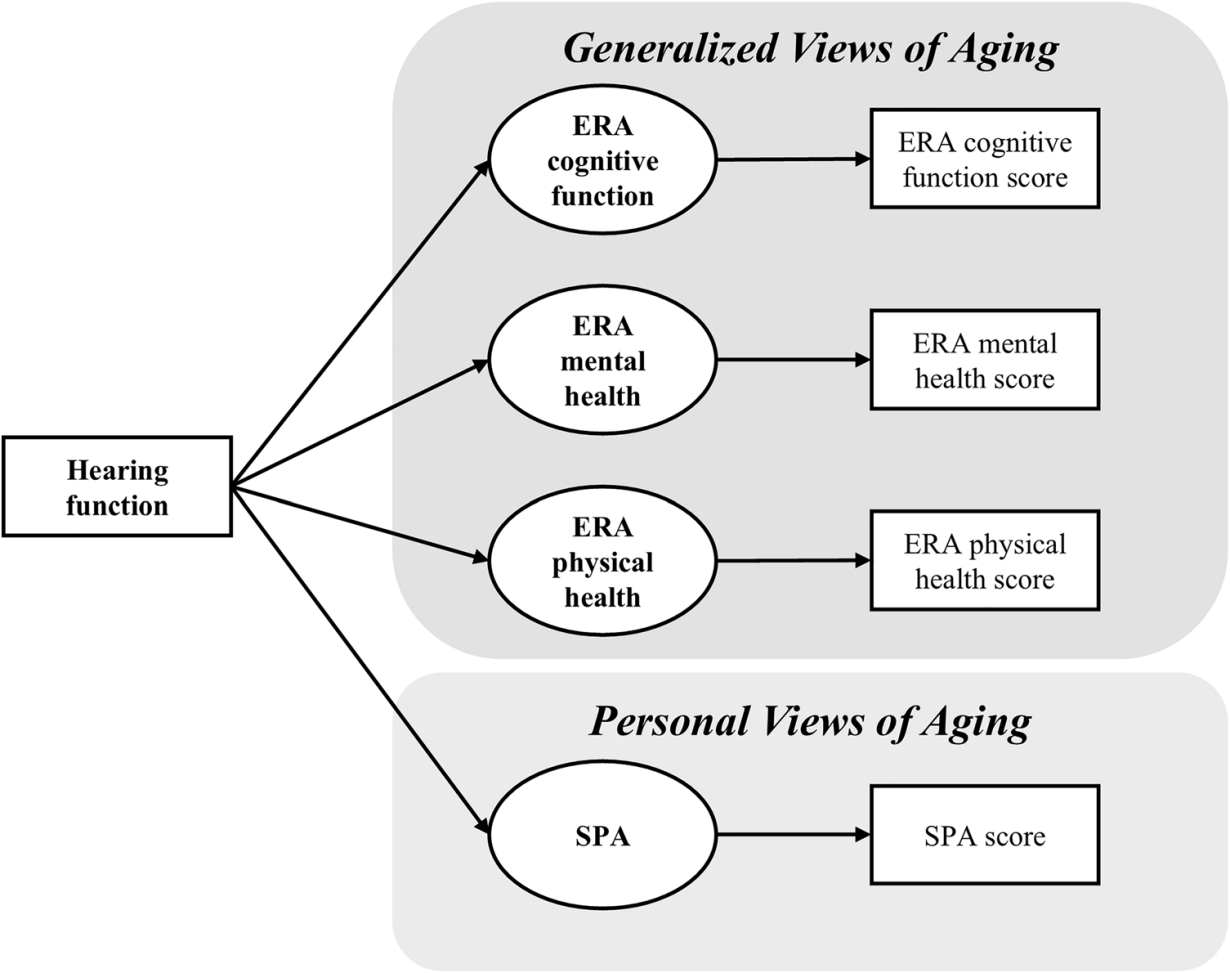
Proposed model of the association between hearing function and different constructs of Views of Aging. Squares represent observed measures, whereas circles represent latent variables. ERA = Expectations regarding aging; SPA = Self-perceptions of aging

**Fig. 2:**
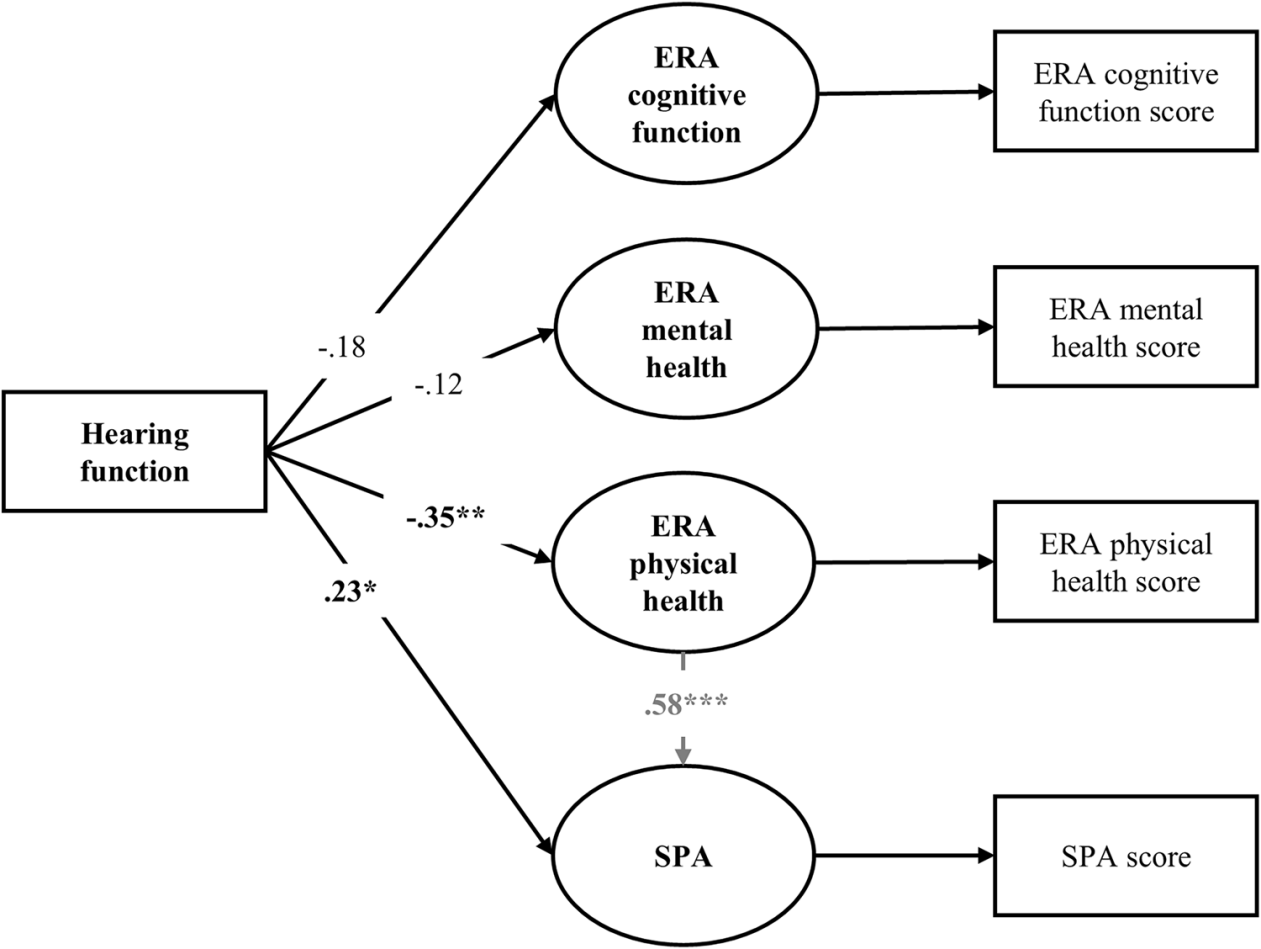
Final SEM model with standardized coefficients. For visual clarity, sociodemographic control variables and other model values (e.g., error variances and factor loadings) were omitted from this figure. A full model with all variables and values is included in the supplementary material (Figures S1 and S2). *p < 0.05. **p < 0.01

Model fit statistics for our proposed model (Fig. 1) and our final model (Fig. 2) are presented in Table 3. Modification indices suggested that the initial model could be significantly improved by considering the following additional relations: (1) We included covariances between the expectations regarding aging subscales, as they underlie the overarching ERA scale. (2) We included one direct path from ERA physical health to SPA, as it is theoretically justified that generalized VoA influence personal VoA (Levy 2009). For our final SEM model, adjusted for chronological age (and interaction term with PTA), sex, loneliness, and cognition, see Fig. 2 (and Supplementary Figure S2).

**Table 3:** Model fit indices

	﻿χ^2^	*df*	RMSEA	SRMR	Tucker–Lewis	CFI
Proposed model	119.2	22	.173	.08	.518	.807
Final model	16.7	18	.000	.02	1.008	1.000

The proposed model included direct pathways from hearing function (PTA) to ERA mental health, ERA physical health, ERA cognitive function, SPA. Control variables were covarianced with the predictor variable (PTA), and had direct pathways to ERA mental health, ERA physical health, ERA cognitive function, and SPA. The final model retained all pathways from the proposed model but also included covariances between the three ERA subscales and one additional direct path from ERA physical health to SPA. ﻿χ^2^ = chi-square; df = degrees of freedom; RMSEA = root mean square error of approximation; SRMR = standardized mean square residual; CFI = comparative fit index; PTA = pure-tone average; ERA = Expectations regarding aging; SPA = Self-perceptions of aging

### Evaluation of the final structural equation model

Model fit statistics indicated that the final model yielded an adequate fit (χ^2^= 16.7; df = 18 (*p* = 0.547), RMSEA = 0.000, SRMR = 0.02, Tucker–Lewis Index = 1.008, CFI = 1.000). Figure 2 displays the standardized regression weights for the hypothesized paths in the model (for unstandardized results, refer to Supplementary Table S2). In sum, after controlling for covariates, poorer hearing function was observed to be significantly associated with worse expectations regarding aging physical health (*β* =  − 0.35, *p* = 0.002) and negative self-perceptions of aging (*β* = 0.23, *p* = 0.016). However, there were no significant associations between hearing function and expectations regarding mental health (*β* =  − 0.12, *p* = 0.264), or cognitive function (*β* =  − 0.18, *p* = 0.110). The interaction term investigating a potential age moderation effect was not significant in predicting expectations regarding aging or self-perceptions of aging. The additional pathway from ERA physical health to SPA was significant (*β* = 0.579, *p* < 0.001). For a detailed overview of regression weights between control variables and main study variables of interest refer to Supplementary Table S3. Together with all control variables, hearing function explained 19% of variance in expectations regarding physical health, 17% in expectations regarding cognitive function, 35% in expectations regarding mental health, and 61% in self-perceptions of aging.

## Discussion

This study investigated how objectively measured hearing function is associated with expectations regarding aging and self-perceptions of aging, while controlling for sociodemographic factors. Cross-sectionally, individuals with poorer hearing exhibited more negative expectations about maintaining physical health as well as more negative self-perceptions of aging compared to those with better hearing. Contrary to our hypotheses, we found no evidence of associations between poor hearing and expectations regarding mental health or cognitive function. Overall, our findings highlight the importance of distinguishing between different constructs of VoA by demonstrating that objective hearing function relates to only two of the four measured constructs.

To date, literature has primarily focused on the relationship between hearing loss and personal VoA (Jang et al. 2004; Kim et al. 2012; Nakagawa et al. 2018; Schroyen et al. 2020; Wettstein et al. 2023b). Our findings align with previous research (Nakagawa et al. 2018; Schroyen et al. 2020; Wettstein et al. 2023b), but contrast with others (Jang et al. 2004; Kim et al. 2012; Wettstein et al. 2023a), suggesting that a poorer hearing function is associated with more negative personal VoA, specifically, self-perceptions of aging. A key distinction in our study is the inclusion of participants aged 40 and older, whereas most previous studies focused on those aged 60 and above. This broader age range may explain the significant association we observed between hearing function and SPA, as younger individuals might perceive hearing loss as unexpected or premature for their age, thereby negatively influencing their self-perceptions of aging.

However, despite this potential age-related difference in how hearing difficulties are perceived, the interaction between age and PTA was non-significant, indicating that age did not statistically moderate the association between hearing and SPA. Two possible explanations may account for this unexpected finding: First, although our sample size was sufficient for the proposed SEM model, it may have been on the threshold of detecting statistical significance for the interaction term (Aguinis et al. 2013). Second, most participants in the younger age group had mild hearing difficulties, which may have resulted in limited variability in hearing levels across age groups. Importantly, while our results did not provide statistical evidence for age as a moderator, they do not necessarily rule out meaningful age-related differences in the effect of hearing loss on SPA. As Knol & VanderWeele (2012) highlight, statistical interaction and effect measure modification are distinct concepts, and stratified analyses can provide further insights into potential subgroup differences. However, due to our sample size constraints, stratification was not feasible in our current study. Future research with larger sample sizes is needed to determine whether hearing loss affects VoA differently across age groups and whether these differences represent true effect modification or statistical interaction (Knol & VanderWeele 2012).

Another notable difference from most aforementioned studies is our use of an objective hearing task in place of a self-rated hearing question. Prior research has indicated that self-rated health status tends to have a stronger association with self-perceptions of aging than objective health parameters (Spuling et al. 2013), as self-perceptions of aging often reflect an awareness of health-related changes (Diehl & Wahl 2024). However, our study provides novel insights by demonstrating that objectively measured hearing function is also associated with VoA. It is not uncommon for individuals with hearing difficulties to be in denial of their condition, making them less aware of how it might actually impact their everyday lives and relationships. Objective measures of hearing function are recognized to offer greater accuracy than self-rated measures (Tsimpida et al. 2020). Consequently, our findings underscore the importance of early detection and intervention of hearing loss as a potential strategy in tackling not only key well-being factors, but also the adverse impact of hearing loss on negative age views.

The current research extends the literature by showing that hearing loss is also associated with generalized VoA, demonstrating that it influences not only individuals’ perceptions of their own aging but also broader age views and expectations about maintaining physical health in later life. The association between hearing and age expectations about physical health may indicate that people with hearing difficulties rate and expect their overall physical health to be more negative due to the significant impact of hearing difficulties on daily life and activities. It is plausible that hearing loss is seen as a reminder of aging, thereby serving as a confirmation of existing age stereotypes related to frailty and physical impairments (Kornadt & Rothermund 2011). Relatedly, the stereotype-matching effect suggests that stereotypes about aging can have a more significant impact on physical health if they match people’s own experiences (Levy & Leifheit-Limson, 2009). Although cognitive decline is a common stereotype of old age (Kornadt & Rothermund 2011), our study found no evidence that individuals experiencing hearing loss expect their cognitive health to decline. This might suggest that people tend to view hearing loss as a physical decline, separate from their concerns about maintaining cognitive health in later life. Similarly, the association of hearing function with expectations regarding mental health was insignificant, possibly due to fewer stereotypes linking aging with mental health (Kornadt & Rothermund 2011). Our study showed that despite a non-significant relationship with hearing, there was a moderate amount of variance explained in expectations regarding mental health. This suggests that the control variables considered in this study are particularly influential. In fact, loneliness was strongly associated with expectations regarding mental health, as well as all other VoA constructs. While not a primary focus of this study, these findings suggest loneliness could play a moderating role between hearing and VoA, particularly expectations regarding mental health. Given that hearing loss often contributes to social isolation due to communication difficulties and the social stigma surrounding it, individuals with hearing loss often experience heightened feelings of loneliness (Shukla et al. 2020). In turn, loneliness has been strongly linked to poorer mental health outcomes, such as depression and anxiety. It is plausible that hearing loss does not directly shape negative expectations about future mental health but contributes to them indirectly through its association with loneliness. However, future research is needed to confirm this hypothesis.

### Practical implications and future directions

In line with aging and human development theories (Diehl et al. 2014; Levy 2009), our findings reinforce that generalized VoA play a key role in shaping personal VoA. Unlike previous studies, which have largely overlooked generalized VoA, our model highlighted how hearing loss influences self-perceptions of aging through its impact on age expectations. If the results of the present study are replicated, addressing age expectations may be an important target for interventions aimed at improving well-being and quality of life among individuals with hearing loss. In that vein, education is arguably the most effective approach in tackling negative age views, as demonstrated, for example, by the AgingPLUS trial in the U.S. (Nehrkorn-Bailey et al. 2023). This 8-week intervention, including educational sessions about healthy aging, the impact of negative views of aging, and physical activity, improved negative expectations regarding aging (as measured on the ERA scale) (Nehrkorn-Bailey et al. 2023). In the context of hearing loss, incorporating educational sessions on hearing health into interventions for healthy aging could be essential. These sessions could focus on the importance of maintaining ear health, early hearing loss detection and correction by regular hearing check-ups, and adequate use of hearing aids. Such education could be extended not only to individuals currently managing hearing loss, but also the general population, as recent research has highlighted a general lack of awareness and knowledge surrounding the broader health implication of hearing loss (Carlson et al. 2022). Furthermore, educating the public about hearing health and the advances in hearing aid technology could help address the social stigma surrounding hearing loss. We also highlight the opportunity for future research to explore the interrelationships among age views, hearing health-related behaviors, and cognitive outcomes for people with HL. For example, future research would benefit from examining the bi-directional relationship between positive VoA and more frequent hearing aid use among older adults. As previous research has linked improved health-related behaviors, such as hearing aid use, to better cognitive outcomes over time (Lin et al., 2023), additional insights into these connections would create opportunities for targeted interventions aimed at promoting healthy (brain) aging.

Our findings support the importance of multidimensional measures of VoA by demonstrating that hearing was associated with only one domain of ERA. This suggests that hearing loss may impact only specific aspects of the aging experience—a hypothesis also supported by Wettstein and colleagues in their research on hearing loss and domain-specific awareness of age-related change (Wettstein et al. 2023b). Moving forward, more research exploring the relationships between hearing function and domain-specific VoA is required. Qualitative research exploring the complexity of these relationships as they are experienced by people with hearing loss may be particularly fruitful for guiding future research directions in this space. Additionally, the growing emphasis on both gain-related and loss-related aspects of aging raises important considerations regarding how VoA is measured. The ERA scale, for example, consists entirely of negatively worded items, which might lead to priming negative age expectations and contribute to an overrepresentation of decline-focused views. As the field evolves, there is an opportunity to develop or incorporate more balanced measures that capture both positive and negative expectations of aging.

Future research could investigate additional factors that may influence the relationship between hearing and VoA. For example, our sample lacked cultural diversity that may be important in the relationships between hearing and VoA. Nakagawa and colleagues conducted the only other study that reported some significant results between hearing and SPA while investigating two culturally different subsamples (US vs Japan) (Nakagawa et al. 2018). While their study did not specifically focus on cultural–societal influences of SPA, previous other studies have documented a relationship between culture and SPA (for example: Wolff et al. 2017). Although we did collect data on cultural background, the limited cultural diversity within our sample made additional subgroup analyses statistically not feasible. Similarly, lifestyle factors, chronic conditions, or overall health status could have influenced the associations between hearing function and VoA. However, due to the design of the study, we were unable to capture or incorporate these factors. This limitation introduces the possibility for unmeasured confounding or differential results. It is likely that hearing loss was not the only health condition contributing to the shaping of VoA; but rather, an accumulation of health-related events or conditions (Rupprecht et al. 2022). Future research could extend the work of Schönstein and colleagues, who investigated multiple health conditions as antecedents as VoA (Schönstein et al. 2021). Finally, exploring the discordance between self-rated and objective hearing within one study could provide further insights into how differing perceptions of hearing influence VoA.

### Strengths and limitations

Notable strengths of this study include the broad age range of the sample, the measurement of both generalized and personal VoA, as well as the use of an objective measure of hearing. Yet, it is important to interpret findings with caution due to some limitations. Firstly, our results are cross-sectional; therefore, the direction of the associations should be interpreted carefully as a bi-directional influence of hearing and VoA is plausible. Nonetheless, our findings suggest that investigating these associations, particularly age expectations about physical health and self-perceptions of aging, in a longitudinal study that can consider the bi-directionality of these variables could be a fruitful avenue for future research. Our sample was somewhat selective, with only iPhone users eligible to participate. This might have led to a sample with higher-than-average health and technological literacy, which could limit the generalizability of the findings to populations less familiar and comfortable with technology or health management. However, while our sample might not fully reflect the broader population in terms of technological and health literacy, it is representative of the general Australian population in terms of hearing loss prevalence (Department of Health and Aged Care, 2022; Wang et al. 2019). Furthermore, our sample was limited by the number of people who reported significant hearing impairment. Future research with a larger sample size would allow for the exploration of the relationships between hearing and VoA with greater differentiation between those experiencing mild, moderate, severe, and profound hearing loss. We note that participants were instructed to complete the app-based hearing task using a set of wired EarPods provided by the research team. We did not assess compliance among people who usually wear hearing aids. Future research would benefit from device recognition coding that could ensure accurate record keeping regarding the accessories used to complete remote hearing assessments. We did not collect data on the number of participants who usually wear hearing aids or the typical duration of their use. As our data reflects uncorrected hearing, it might not fully represent the everyday sensory–perceptual experiences of all participants. However, given that many individuals who would benefit from hearing aids either do not use them at all, or wear them inconsistently (Knoetze et al. 2023), our data likely remains broadly representative. Future research could examine whether hearing aid use moderates the relationship between (uncorrected) hearing loss and VoA, as this could further highlight the importance of promoting hearing aid adoption. It is also noteworthy that the hearing data in this study was collected three to seven weeks after collection of the other study variables. Although some research indicates that personal VoA can fluctuate to a small degree on a day-to-day basis (Kotter-Grühn et al., 2015), they generally tend to remain relatively consistent over time (Rubin & Berntsen 2006). Given the overall stability of all other variables (i.e., age, sex, and hearing function) across short timescales, we do not anticipate that the delay in collection of hearing data will have impacted the results.

### Concluding remarks

Our research reports new insights into the associations between hearing function—a common age-related health decline—and Views of Aging. We demonstrated that poorer hearing is associated with more negative age expectations regarding physical health and negative self-perceptions of aging. Additionally, these findings support existing research on the multidimensionality of subjective aging, while highlighting the importance of distinguishing between different constructs of Views of Aging. If findings are replicated in a longitudinal study, they would further highlight the importance for early detection and intervention of hearing loss, not only to counteract negative social and cognitive outcomes, but also to address adverse effects on VoA.

## Supplementary Information

Supplementary file 1.

## Data Availability

The datasets generated and analyzed during the current study are not publicly available as, according to the approved ethics protocol, participants did not consent to their data being shared outside the immediate research team without ethical approval. However, authors can be contacted with any questions related to the data and de-identified data are available on reasonable request with ethical approval.
