# Supplementary material for: Associations between objective hearing function and subjective views of aging
Source: Eur J Ageing. 2025 Jul 16;22(1):33. doi: 10.1007/s10433-025-00868-8 (PMC12267798; doi:10.1007/s10433-025-00868-8)

Online Supplementary Materials

Associations Between Objective Hearing Function and Subjective Views of Aging

European Journal of Ageing

Jana Koch*, Brooke Brady, Lidan Zheng, Kaarin J. Anstey

*Corresponding author

Email: jana.koch@unsw.edu.au

School of Psychology, University of New South Wales Sydney, Kensington, Australia

Neuroscience Research Australia, Randwick, Australia

UNSW Aging Futures Institute, UNSW Sydney, Kensington, New South Wales, Australia

Figure S1: Final structural equation model for the estimation of the association between hearing function and expectations regarding aging and self-perceptions of aging; all inclusive with sociodemographic control variables displayed.

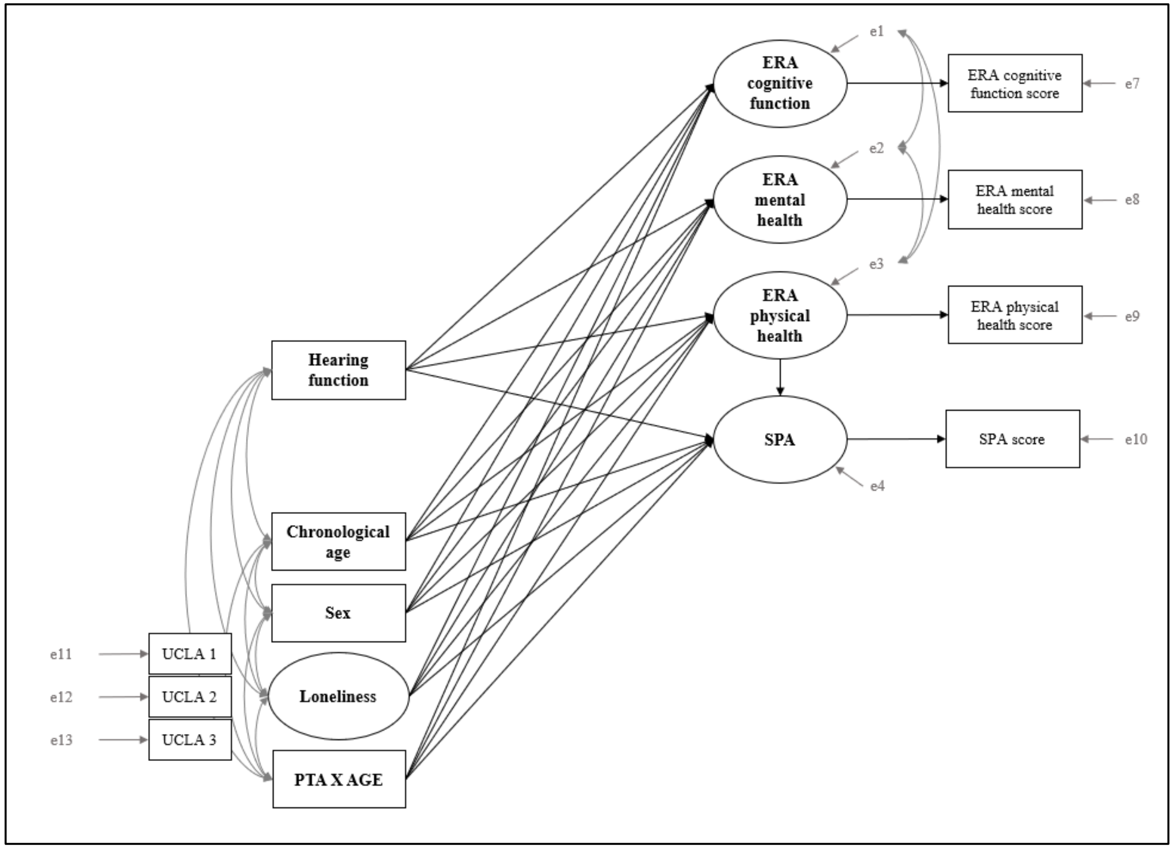

Note. ERA=Expectations regarding aging, SPA=Self-perceptions of aging. PTA=Pure tone average. e=measurement errors.

Figure S2: Full structural equation model with model values. Standardized regression coefficients between control variables and outcome variables are not shown (refer to Table S2). Model fit indices: X^2=16.7, df=18, RMSEA=0.000, SRMR=0.02, Tucker-Lewis=1.008, CFI=1.000.

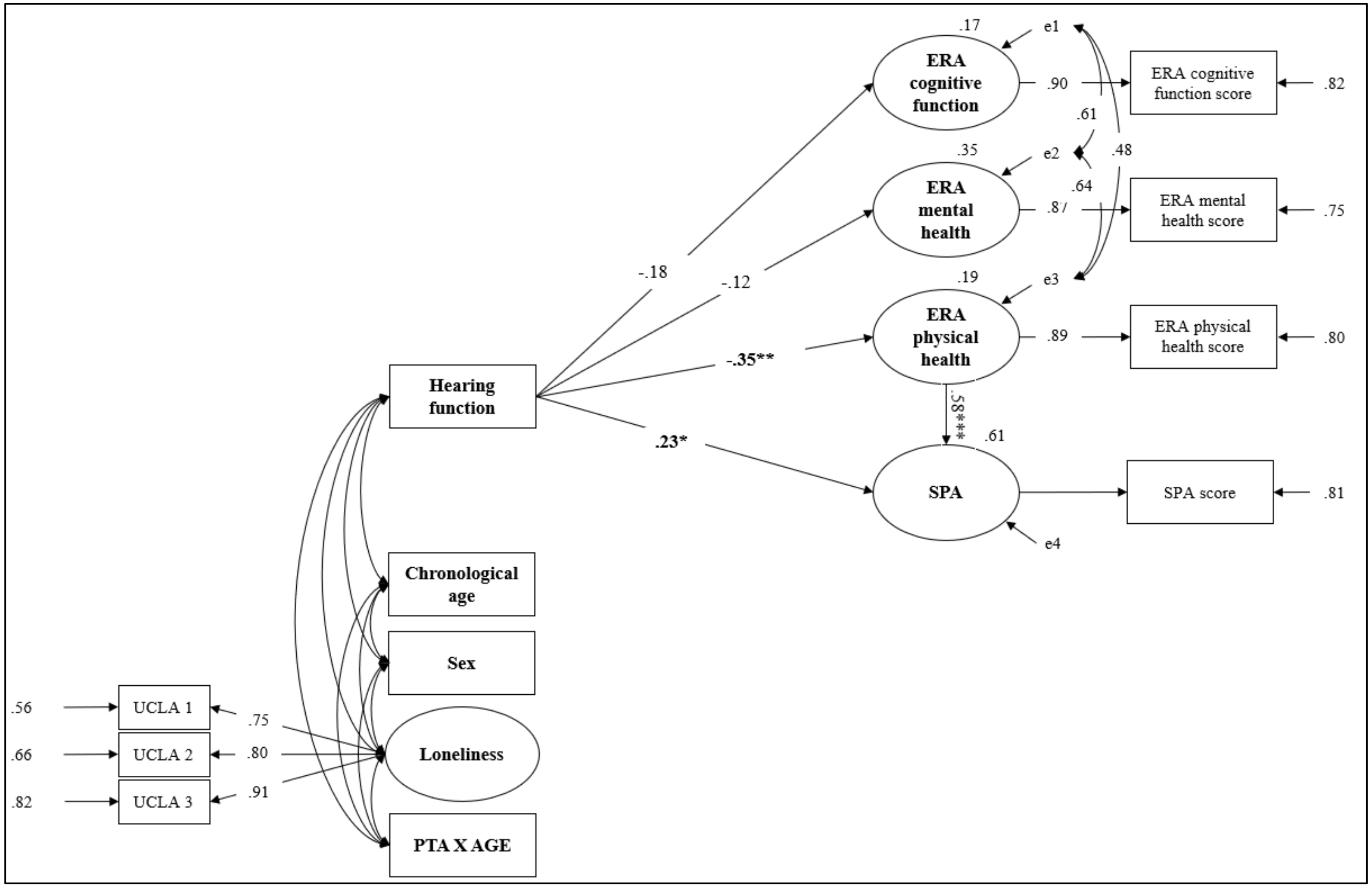

Note. ERA=Expectations regarding aging, SPA=Self-perceptions of aging. e=measurement error. *p<0.05. **p<0.01.***p<0.001.

Table S1: Hearing loss classification based on the World Health Organization (Humes, 2019).

	**WHO grading**	**Grading in the present study**
Normal hearing	<20 dBHL	<20 dBHL
Mild hearing loss	21-34 dBHL	21-34 dBHL
Moderate hearing loss	35-49 dBHL	35-49 dBHL
Moderately severe hearing loss	50-64 dBHL	
Severe	65-79 dBHL	>50 dBHL
Profound impairment	80-91 dBHL	

Note. Due to small sample sizes, moderately severe, severe, and profound impairment categories were combined into a single 'severe HL' category defined by hearing impairment at greater than or equal to 50 dBHL.

WHO=World Health Organization; dBHL=decibles hearing level.

Table S2: Unstandardized regression coefficients of main pathways of interest.

Exogenous parameter		Endogenous parameter	Estimate (B)	Standard Error	p-value	Standardized B
Hearing function			**Generalized VoA**			
	→	ERA, cognitive function	-.014	.009	.110	-.18
	→	ERA, physical health	-.03	.009	**.002**	**-.35**
	→	ERA, mental health	-.010	.009	.264	-.12
			**Personal VoA**			
	→	Self-perceptions of aging	.019	.008	**0.016**	**.23**

Note. ERA=Expectations regarding aging; SPA=Self-perceptions of aging. VoA=Views of Aging.

Table S3: Standardized and unstandardized regression weights of final structural equation model.

Exogenous	Endogenous	Estimate (B)	Standard Error	p-value	Standardized B
Chronological age	→ ERA, cognitive function	-.016	.009	.076	-.182
	→ ERA, mental health	.004	.009	.650	.046
	→ ERA, physical health	.006	.009	.494	.071
	→ SPA	-.011	.008	.136	-.128
Sex	→ ERA, cognitive function	.411	.182	**.024**	.196
	→ ERA, mental health	.711	.173	**<.001**	.349
	→ ERA, physical health	.412	.183	**.025**	.195
	→ SPA	-.064	.152	.675	-.031
Loneliness	→ ERA, cognitive function	-.472	.201	**.019**	-.218
	→ ERA, mental health	-1.039	.199	**<.001**	-.495
	→ ERA, physical health	-.567	.204	**.005**	-.261
	→ SPA	-.905	.179	**<.001**	-.426

PTA X Age	→ ERA, cognitive function	.066	.090	.461	.071
	→ ERA, mental health	.028	.085	.745	.031
	→ ERA, physical health	.046	.090	.607	.049
	→ SPA	-.101	0.73	.167	-.110
ERA, physical health	→ SPA	.566	.088	**<.001**	.579

Note. ERA=Expectations regarding aging; SPA=Self-perceptions of aging. PTA=Pure tone average. Sex was coded as 0=Male and 1=Female.

Figure S3: Flowchart participant inclusion and missing data.

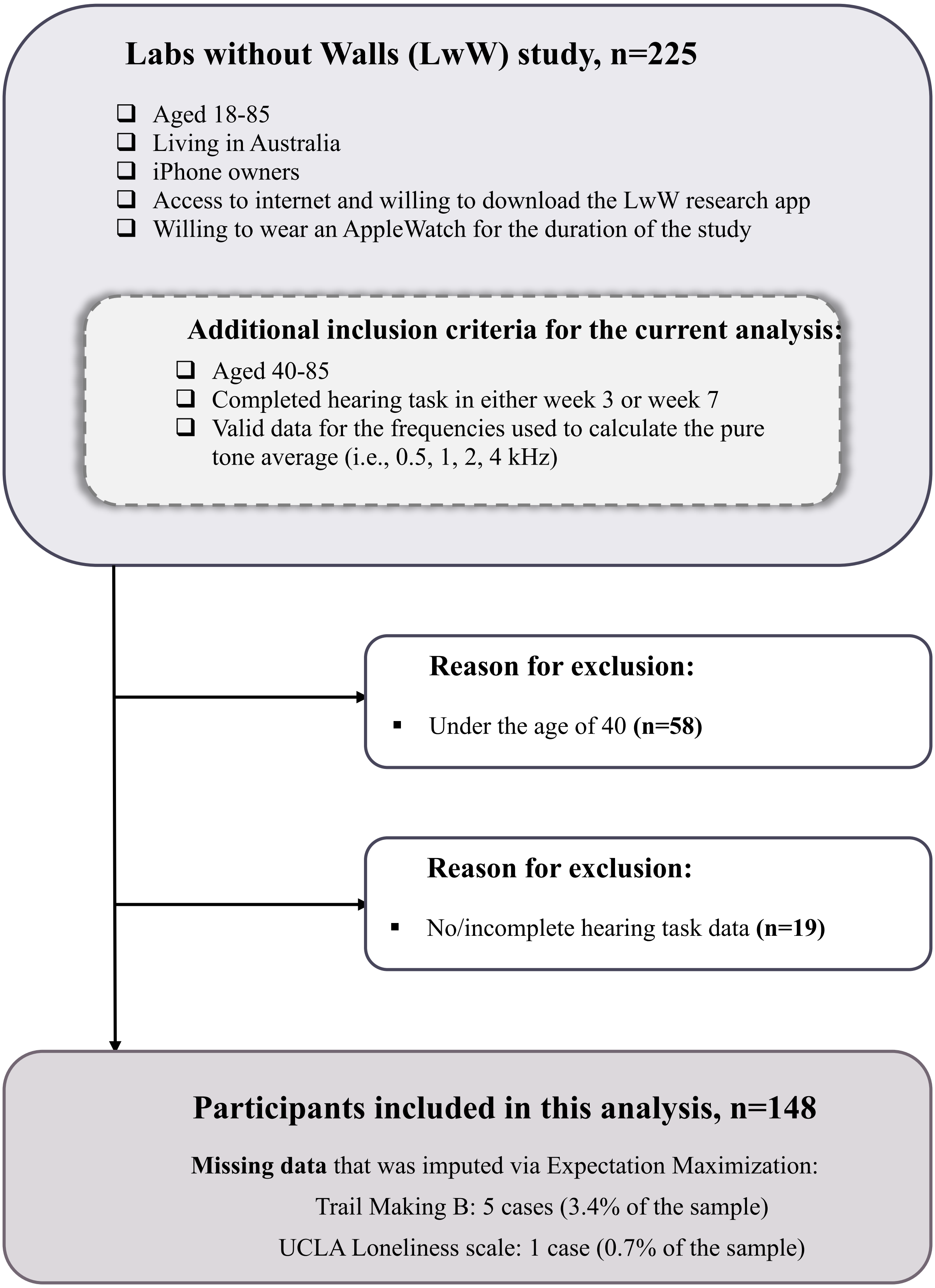

Supplement: Supplementary file 1 [file 10433_2025_868_MOESM1_ESM.pdf]
